# Prognostic factors for progression of clinical osteoarthritis of the knee: a systematic review of observational studies

**DOI:** 10.1186/s13075-015-0670-x

**Published:** 2015-06-08

**Authors:** Alex N. Bastick, Jos Runhaar, Janneke N. Belo, Sita M.A. Bierma-Zeinstra

**Affiliations:** Department of General Practice, Erasmus MC, University Medical Center Rotterdam, PO Box 2040, Rotterdam, 3000 CA The Netherlands; Department of Public Health and Primary Care, Leiden University Medical Center, PO Box 9600, Leiden, 2300 RC The Netherlands

## Abstract

**Introduction:**

We performed a systematic review of prognostic factors for the progression of symptomatic knee osteoarthritis (OA), defined as increase in pain, decline in physical function or total joint replacement.

**Method:**

We searched for available observational studies up to January 2015 in Medline and Embase according to a specified search strategy. Studies that fulfilled our initial inclusion criteria were assessed for methodological quality. Data were extracted and the results were pooled, or if necessary summarized according to a best evidence synthesis.

**Results:**

Of 1,392 articles identified, 30 met the inclusion criteria and 38 determinants were investigated. Pooling was not possible due to large heterogeneity between studies. The best evidence synthesis showed strong evidence that age, ethnicity, body mass index, co-morbidity count, magnetic resonance imaging (MRI)-detected infrapatellar synovitis, joint effusion and baseline OA severity (both radiographic and clinical) are associated with clinical knee OA progression. There was moderate evidence showing that education level, vitality, pain-coping subscale resting, MRI-detected medial femorotibial cartilage loss and general bone marrow lesions are associated with clinical knee OA progression. However, evidence for the majority of determinants was limited (including knee range of motion or markers) or conflicting (including age, gender and joint line tenderness).

**Conclusion:**

Strong evidence was found for multiple prognostic factors for progression of clinical knee OA. A large variety in definitions of clinical knee OA (progression) remains, which makes it impossible to summarize the evidence through meta-analyses. More research on prognostic factors for knee OA is needed using symptom progression as an outcome measure. Remarkably, only few studies have been performed using pain progression as an outcome measure. The pathophysiology of radiographic factors and their relation with symptoms should be further explored.

## Introduction

Osteoarthritis (OA) is one of the most common chronic diseases and is one of the leading causes of pain and disability worldwide. Amongst patients with OA, the incidence and prevalence of knee OA is the highest [1]. Consequently, many studies have been and are being performed to determine prognostic factors for knee OA. Previously, Belo et al. [2] published a systematic review determining all prognostic factors for knee OA. Their literature search was performed up to 2003 and none of the included articles had used clinical outcome measures to assess knee OA progression. An update of the review by Belo et al. [3]. has recently been performed by the same authors, but again only focuses on radiographic progression of knee OA when a clear discordance between radiographic and symptomatic knee OA has formerly been established [4]. Also, symptomatic progression of knee OA is most relevant for the patient and the physician in clinical practice. Therefore, we have chosen to perform a systematic review of prognostic factors for the symptomatic (i.e., clinical) progression of knee OA. To our knowledge, this is the first systematic review of its kind.

## Methods

### Literature search

Our search was performed in Medline and Embase up to January 2015. The keywords used were: knee, osteoarthritis (or arthritis, or arthrosis, or degenerative joint disease), progression (or prognosis, or precipitate, or predictive), clinical (or symptomatic) and case–control (or cohort, or longitudinal, or follow-up). All abstracts and, if necessary, full texts of the identified references were reviewed for inclusion independently by two authors (ANB and JR or JNB). The following inclusion criteria were used: ≥85 % of the patients used in the analyses for OA progression had clinical (i.e., American College of Rheumatology (ACR) or Osteoarthritis Research Society International Atlas (OARSI) criteria) or radiographic evidence of knee OA at baseline (equivalent to a Kellgren and Lawrence (K/L) score ≥2 at baseline); the study investigated determinants associated with the clinical progression of knee OA; a specific clinical outcome measure was appointed, i.e., pain, function or knee joint replacement; the study had either a case–control or cohort design with a minimal follow-up period of 1 year; the full text of the article was available; and the study was written in English, Dutch, German or French. Studies that merely observed incidence of knee OA were excluded. Studies determining magnetic resonance imaging (MRI) features as prognostic factors were included as long as a clinical outcome measure was applied. Another reason for exclusion was if the study population had an underlying pathology (e.g., rheumatoid arthritis, bacterial infection) of the joint. Finally, inclusion of articles was extended if a relevant article was detected when screening the references of included articles.

### Methodological quality

The methodological quality assessment criteria were based on previously described criteria by Lievense et al. [5], Scholten-Peeters et al. [6], and Altman [7] (Table 1). All included articles were scored independently by two authors (ANB and JR or JNB) with a maximum score of 13 points. In case of disagreement, the authors arranged an appointment to achieve consensus. Noteworthy is that we only scored the articles based on the data that were published in the manuscripts; hence, characteristics of the selected population under study that were published elsewhere were not incorporated in the quality score.

**Table 1:** Methodological quality assessment criteria

Quality criteria	Score
Study population	
A) Description of source population	1
B) Valid inclusion and exclusion criteria	1
C) Sufficient description of baseline characteristics	1
Follow-up	
D) Follow-up of at least 1 year	1
E) Prospective or retrospective data collection	1
F) Loss to follow-up ≤20 %	1
G) Information about loss to follow-up (selective for age, sex or severity)	1
Exposure	
H) Exposure assessment blinded for the outcome	1
I) Exposure measured identically in the studied population at baseline and follow-up	1
Outcome	
J) Outcome assessment blinded for exposure	1
K) Outcome measured identically in the studied population at baseline and follow-up	1
Analysis	
L) Measure of association or measures of variance given	1
M) Adjusted for age, sex and severity	1

### Data extraction

Study population characteristics, observed risk factors, definitions of knee OA progression and measures of association or correlations, including odds ratios (OR), relative risks (RR), hazard ratios (HR) or regression coefficients and their 95 % confidence intervals (CI) were extracted and are presented in this review.

### Evidence synthesis

OR, RR or HR were pooled when clinical homogeneity in the study population, measured determinants and assessed outcome was assumed (using *Review Manager* (*RevMan*). Version 5.3. Copenhagen: The Nordic Cochrane Centre, The Cochrane Collaboration, 2014). In the absence of clinical homogeneity, a best evidence synthesis was used to summarize the data. The level of evidence was based on the updated guidelines by Furlan et al. [8] and was divided into the following levels: A) strong evidence, i.e., consistent (>75 %) findings amongst multiple (≥2) high-quality studies; B) moderate evidence, i.e., findings in one high-quality study and consistent (>75 %) findings in ≥2 low-quality studies; C) limited, evidence, i.e., findings in one high-quality study or consistent findings in ≥3 low-quality studies; and D) conflicting or inconclusive evidence, i.e., <75 % of the studies reported consistent findings, or the results were only based on one study. Articles were scored as high quality when they had a quality score ≥9 (>65 % of the maximal attainable score). Only statistically significant associations were considered as associated prognostic factors in the best evidence synthesis.

### Sensitivity analysis

If we were forced to perform a best evidence synthesis, we conducted a sensitivity analysis to check whether differences in sample size (cut-off N = 200) could have altered our conclusions. Additionally we checked whether large variances in follow-up (cut-off 24 months) duration could have led to different conclusions. Finally, we checked whether our conclusions could have been influenced by differences in definitions for clinical OA (cOA) progression in the included articles; for instance, knee joint replacement as opposed to pain progression or function decline.

## Results

### Studies included

Of the 1,392 articles identified using our search strategy, 30 articles met the inclusion criteria [9–38]. Three reviewers scored a total of 390 items for the methodological quality assessment and agreed on 351 items (90 %; κ 0.71). The 39 disagreements were resolved in a single consensus meeting.

Of the 30 articles 20 were of high quality and scored in the range of 9–13. Almost all studies had a prospective research design. Three definitions of OA were used for the inclusion of participants: 17 studies used the K/L criteria, 11 articles applied the ACR criteria and 2 studies used the OARSI. Four of the studied populations contained more men than women, all other studies contained more women. A full overview of these results, including study sample sizes and follow-up durations, is presented in Table 2. Fifteen different definitions were used to define progression of cOA, including knee joint replacement, symptom severity on the Western Ontario and McMasters osteoarthritis index (WOMAC) scales for pain, function or stiffness and visual analogue scale (VAS) for pain. The definitions for cOA progression are presented in the corresponding tables which are discussed below.

**Table 2:** Study characteristics of the included studies (n = 30)

Author [ref.] year	Follow-up months	Definition of OA for inclusion	Age, years	Women, %	No. of patients	Quality score	A	B	C	D	E	F	G	H	I	J	K	L	M
Amin [10] 2009	30	ACR criteria	69	42	265	13	1	1	1	1	1	1	1	1	1	1	1	1	1
Tanamas [35] 2010	24	K/L	63.2 ± 10.3	51	109	13	1	1	1	1	1	1	1	1	1	1	1	1	1
Cicuttini [14] 2004	24	K/L	63.1 ± 10.3	58	113	12	1	1	1	1	1	1	0	1	1	1	1	1	1
Hill [19] 2007	30	ACR criteria	66.7 ± 9.2	41	233	12	1	1	1	1	1	1	0	1	1	1	1	1	1
Holla [21] 2014	60	ACR criteria	56.0 ± 5.1	81.3	697	12	1	1	1	1	1	1	1	0	1	1	1	1	1
Tanamas [36] 2010	24	ACR criteria	63.2 ± 10.3	70	109	12	1	1	1	1	1	1	1	0	1	1	1	1	1
Berry [12] 2010	24	ACR criteria	63.7 ± 10.3	58	117	11	1	1	1	1	1	1	1	0	1	0	1	1	1
Henriksen [18] 2013	12	K/L	63	82	157	11	1	0	1	1	1	1	1	1	1	0	1	1	1
Yang [38] 2015	36	K/L	43 % >65	58	1,625	11	1	0	1	1	1	1	0	1	1	1	1	1	1
Alschuler [9] 2013	12	K/L	65.3 ± 9.0	59	797	10	1	1	1	1	1	0	0	0	1	1	1	1	1
Amin [11] 2008	30	ACR criteria	67 ± 9	43	265	10	1	1	1	1	1	1	1	0	1	0	1	1	1
Collins [15] 2014	72	K/L	62 ± 9	59	1,753	10	1	0	1	1	1	0	0	1	1	1	1	1	1
Holla [20] 2010	24	ACR criteria	56.0 ± 5.1	80	832	10	1	1	1	1	1	1	1	0	1	0	1	1	1
Lapane [22] 2015	48	K/L	70	58	1,846	10	1	0	1	1	1	0	0	1	1	1	1	1	1
Larsson [23] 2012	90	OARSI	50 (32–73)	18	74	10	1	1	1	1	1	1	0	0	1	1	1	1	1
Laslett [24] 2014	60	K/L	61	100	323	10	1	1	1	1	1	0	0	1	1	0	1	1	1
Muraki [25] 2012	40	K/L	68.7 ± 11.3	75	1,313	10	1	1	1	1	1	1	0	0	1	1	1	1	1
Podsiadlo [28] 2014	72	ACR criteria	63.9	57	114	10	1	0	1	1	1	1	1	0	1	0	1	1	1
Riddle [30] 2012	48	OARSI	62	58	4,670	10	1	1	1	1	1	1	1	0	1	0	1	1	1
Roemer [32] 2014	60	K/L	64.2 ± 8.4	58	398	10	1	1	1	1	1	0	0	1	1	1	1	1	0
Bruyere [13] 2005	45.6	ACR criteria	64.7 ± 7.0	70	139	9	1	0	1	1	1	1	1	0	1	0	1	1	0
Conaghan [16] 2010	36	K/L	67 ± 10	73	531	9	1	1	1	1	1	1	0	0	1	0	1	1	0
Sharma [34] 2003	36	K/L	68.6 ± 10.8	73	236	9	1	1	1	1	1	1	0	0	1	0	1	1	0
Eckstein [17] 2013	48	K/L	58	64	97	8	1	0	1	1	1	0	1	0	1	1	1	0	0
Oak [26] 2013	48	K/L	61.2 ± 9.1	53	942	8	1	0	1	1	1	0	0	0	1	0	1	1	1
Riddle [29] 2009	24	K/L	61.6 ± 9.3	60	778	8	1	1	1	1	1	0	0	0	1	0	1	1	0
Scher [33] 2008	36	K/L	51	63	73	8	1	0	0	1	1	1	0	0	1	1	1	1	0
van Dijk [37] 2011	36	ACR criteria	65.9 ± 8.3	74	174	8	1	0	1	1	1	1	0	0	1	0	1	1	0
Pisters [27] 2012	60	ACR criteria	66.1 ± 8.5	74	216	7	1	0	1	1	1	0	0	0	1	0	1	1	0
Riddle [31] 2013	33	K/L	62.7 ± 8.6	63	1,410	7	1	0	1	1	1	0	0	0	1	0	1	1	0

A–M represent criteria scores (see Table 1). *ACR* American College of Rheumatology, *K/L* Kellgren and Lawrence score, *OA*, osteoarthritis, *OARSI* Osteoarthritis Research Society International Atla

### Study results

We obtained 38 different determinants. We grouped our findings into two pragmatically chosen categories: patient characteristics and disease characteristics. A full overview of the determinants and their potential associations to clinical knee OA progression are presented in Tables 3 and 4. Some authors reported statistically significant associations to OA progression, but used *p* values as indications of association. We chose to only present OR, RR, HR or regression coefficients as measures of associations in our tables, but we have tabulated whether there was a significant association found in an article or not. All measures of association were eventually included in the evidence syntheses.

**Table 3:** Patient characteristics studied as determinants for clinical knee OA progression in the included studies

Determinant	Author [ref.] year	Instrument of measurement	Definition of knee OA progression	OR/RR/HR/β (95 % CI)	Association with OA prognosis*
Age	Muraki [25] 2012	Per 5 years increase	Incident knee pain at follow-up (baseline K/L ≥2)	OR 1.01 (0.95–1.07)	o
(N = 10,043)	Holla [20] 2010	Continuous (years)	Progressing/remaining in poor WOMAC-PF quintiles	OR 0.97 (0.94–1.00)	–
	Holla [21] 2014	Continuous (years)	Poor vs good outcome WOMAC-PF trajectory	OR 0.94 (0.88–1.00)	–
	Collins [15] 2014	Continuous (years)	Severe vs no pain WOMAC pain trajectory	OR 0.92 (0.89–0.96)	–
	Riddle [29] 2009	Continuous (years)	Knee joint surgery	OR 1.07 (1.02–1.11)	+
	Riddle [30] 2012	Continuous (years)	Knee joint surgery	RR 1.04 (1.01–1.23)	+
Female sex	Collins [15] 2014	Female vs male	Severe vs no pain WOMAC pain trajectory	OR 3.0 (1.5–6.2)	+
(N = 3,066)	Muraki [25] 2012	Female vs male	Incident knee pain at follow-up (baseline K/L ≥2)	OR 1.32 (0.94–1.84)	o
Ethnicity	Collins [15] 2014	Non-white vs white	Severe vs no pain WOMAC pain trajectory	OR 3.3 (1.7–6.6)	+
(N = 2,585)	Holla [20] 2010	Non-western vs western	Progressing/remaining in poor WOMAC-PF quintiles	OR 4.03 (1.06–15.4)	+
Education level	Collins [15] 2014	<College ≥ college	Severe vs no pain WOMAC pain trajectory	OR 5.1 (2.3–11.2)	+
(N = 2,531)	Riddle [29] 2009	≤ High school graduate	Knee joint surgery	OR 2.40 (1.09–5.28)	+
Body mass index (N = 4,857)	Holla [20] 2010	Continuous	Progressing/remaining in poor WOMAC-PF quintiles	OR 1.06 (1.02–1.11)	+
	Sharma [34] 2003	Per 5 unit increase	Progressing/remaining in poor WOMAC-PF quintiles	OR 1.15 (0.89–1.46)	o
	Collins [15] 2014	Obese vs non-obese	Severe vs no pain WOMAC pain trajectory	OR 2.3 (1.2–4.4)	+
	Holla [21] 2014	Continuous	Moderate vs good outcome WOMAC-PF trajectory	OR 1.12 (1.07–1.18)	+
	Muraki [25] 2012	Per 5 units increase	Incident knee pain at follow-up (baseline K/L ≥2)	OR 1.54 (1.90–1.82)	+
	Riddle [29] 2009	≤30 vs >30 kg/m^2^	Knee joint surgery	OR 2.66 (1.20–5.92)	+
Bodyweight change	Riddle [31] 2013	≥–10 % vs –4.9 to +4.9 %		β 4.07 (1.49–6.65)	–
(N = 1,410)		–9.9 to –5 % vs –4.9 to +4.9 %	Increase self-reported limitations (WOMAC-PF)	β 0.01 (–1.87 to 1.89)	o
		+5 to +9.9 % vs –4.9 to +4.9 %	Increase in pain (WOMAC); similar results, not tabulated	β 1.08 (–0.91 to 3.07)	o
		≥ + 10 % vs –4.9 to +4.9 %		β –5.36 (–8.74 to –2.00)	+
Knee compression force	Henriksen [18] 2013	Change in peak knee joint compressive force	Change in KOOS-4	LSMD –2.4 (–6.8 to 1.9)	o
(N = 157)			Change in walking speed, m/s	LSMD –0.01 (–0.05 to 0.03)	o
Co-morbidity count	Holla [20] 2010	≥3 vs none	Progressing/remaining in poor WOMAC-PF quintiles	OR 1.53 (0.93–2.53)	o
(N = 3,672)	Holla [21] 2014	≥3 vs < 3	Poor vs good outcome WOMAC-PF trajectory	OR 3.28 (1.62–6.64)	+
	Collins [15] 2014	≥1 vs 0	Severe vs no pain WOMAC pain trajectory	OR 2.0 (1.0–3.9)	+
	Pisters [27] 2012	Per unit CIRS increase	Increase in self-reported limitations (WOMAC-PF)	β 3.69 (1.66–8.23)	+
	Van Dijk [37] 2011	Per unit CIRS increase	Increase in self-reported limitations (WOMAC-PF)	β –0.147 (not provided)	+
			Increase in performance-based limitations (TWT)	β 0.150 (not provided)	+
Mental health	Collins [15] 2014	CES-D ≥16 vs CES-D < 16	Severe vs no pain WOMAC pain trajectory	OR 8.8 (3.1–25.2)	+
(N = 6,659)	Sharma [34] 2003	Per 5 points score	Progressing/remaining in poor WOMAC-PF quintiles	OR 0.58 (0.39–0.86)	–
	Riddle [30] 2012	Per unit SF-12 MCS score	Knee joint surgery	RR 1.07 (1.04–1.10)	+
Vitality	Holla [21] 2014	Per unit SF-36 HS score	Poor vs good outcome WOMAC-PF trajectory	OR 0.96 (0.94–0.98)	–
(N = 871)	Van Dijk [37] 2011	Per unit SF-36 MOS score	Increase in self-reported limitations (WOMAC-PF)	β 0.157 (not provided)	–
			Increase in performance-based limitations (TWT)	β –0.229 (not provided)	–
Pain coping	Alschuler [9] 2013	CSQ subscale praying or hoping	≥20 % change in combined NRS and WOMAC-PF score	Not provided	+
(N = 1,048)		CSQ subscale catastrophizing	≥20 % change in combined NRS or WOMAC-PF score	Not provided	+
	Holla [20] 2010	PCI subscale distraction	Progressing/remaining in poor WOMAC-PF quintiles	OR 1.26 (0.98–1.62)	o
		PCI subscale worrying		OR 0.63 (0.66–0.73)	–
	Holla [21] 2014	PCI subscale resting	Poor vs good outcome WOMAC-PF trajectory	OR 1.16 (1.02–1.31)	+
	Pisters [27] 2012	PCI subscale resting	Increase in self-reported limitations (WOMAC-PF)	β 23.3 (1.93–280.7)	+
			Increase in performance-based limitations (TWT)	β 3.13 (1.95–5.03)	+
Morning stiffness	Holla [20] 2010	<30 minutes, yes vs no	Progressing/remaining in poor WOMAC-PF quintiles	OR 1.37 (0.99–1.88)	o
(N = 832)					
Knee injury	Muraki [25] 2012	Previous knee injury	Incident knee pain at follow-up (baseline K/L ≥2)	OR 2.91 (1.26–6.82)	+
(N = 1,313)					
Knee surgery	Riddle [30] 2012	History of knee surgery	Knee joint surgery	RR 2.04 (1.33–3.13)	+
(N = 4,670)					
Pain medication use	Riddle [30] 2012	For pain, aching or stiffness	Knee joint surgery	RR 1.64 (0.87–3.12)	o
(N = 6,516)	Lapane [22] 2015	NSAID usage vs not	MICD of WOMAC pain	β –0.88 (–2.22 to 0.46)	o
			MICD of WOMAC-PF	β –4.27 (–8.84 to 0.31)	o
			MICD of WOMAC stiffness	β –0.72 (–1.56 to 0.12)	o
Bisphosphonate use	Laslett [24] 2014	Yes vs no	Decrease in WOMAC pain	β 0.69 (–0.54 to 1.92)	o
(N = 323)			Decrease in WOMAC function	β 0.05 (–3.85 to 3.95)	o
			Decrease in WOMAC stiffness	β –0.24 (–0.75 to 0.27)	o
			Decrease in NRS after 3 and 4 years, but not 5 years	β –1.15 (–1.94 to –0.36)	+
Glucosamine/chondroitin use	Yang [38] 2015	Yes vs no	MICD of WOMAC pain	β 0.68 (–0.16 to 1.53)	o
			MICD of WOMAC-PF	β 1.28 (–1.23 to 3.79)	o
(N = 1,625)			MICD of WOMAC stiffness	β 0.41 (0–0.82)	o
History of HRS	Riddle [30] 2012	Yes vs no	Knee joint surgery	RR 2.73 (0.93–8.07)	o
(N = 4,670)					

*Statistically significant association of the determinant with OA progression: + positive association, – negative association, o no association (adjusted for age and sex if applicable).

*β* regression coefficient, *CES-D* Center for Epidemiologic Studies Depression scale, *CI* confidence interval, *CIRS* Cumulative Illness Rating Scale, *CSQ* Coping Strategies Questionnaire, *HR* hazard ratio, *HRS* hip replacement surgery, *K/L* Kellgren and Lawrence score, *KOOS-4* Knee injury and Osteoarthritis Outcome Score for pain, symptoms, function and quality of life, *LSMD* least squares means difference, *MICD* minimally important clinical difference, *N* combined sample size, *NRS* Numeric Rating Scale, *NSAID* non-steroidal anti-inflammatory drug, *OA* osteoarthritis, *OR* odds ratio, *PCI* Pain Coping Inventory, *RR* relative risk, *SF-12 MCS* Short Form survey instrument for the Mental Component Summary, *SF-36 HS/MOS* SF-36 Health Survey/Medical Outcome Study, *TWT* Timed Walking Test, *WOMAC-PF* physical function scale of the Western Ontario and McMaster osteoarthritis index

**Table 4:** Disease characteristics studied as determinants for clinical knee OA progression in the included studies

Determinant	Author [ref.] year	Analysis of determinant	Definition of knee OA progression	OR/RR/HR/β (95 % CI)	Association with OA prognosis*
Severity					
Radiographic	Bruyere [13] 2005	JSN ≥0.5 mm/3 years	Knee joint surgery	RR 4.61 (1.65–12.8)	+
(N = 8,201)	Riddle [30] 2012	Per grade (0–4 grade scale)	Knee joint surgery	RR 2.09 (1.63–2.69)	+
	Collins [15] 2014	K/L 3 vs K/L 2^a^	Severe vs no pain WOMAC pain trajectory	OR 4.3 (2.1–8.6)	+
	Holla [21] 2014	Osteophytosis	Poor vs good outcome WOMAC-PF trajectory	OR 5.68 (2.57–12.55)	+
	Oak [26] 2013	Baseline JSW (mm)	Decrease KOOS pain (KOOS symptom and quality of life show similar regression coefficients)	β 1.94 (1.19–2.69)	+
		JSN over 4 years (mm)		β 2.31 (1.18–3.44)	+
Clinical	Alschuler [9] 2013	NRS of past 7 days^a^	≥20 % change in combined NRS and WOMAC-PF score	Not provided	+
(N = 7,558)		WOMAC-PF^a^		Not provided	+
	Holla [21] 2014	NRS for knee pain^a^	Poor vs good outcome WOMAC-PF trajectory	OR 1.81 (1.51–2.16)	+
	Oak [26] 2013	Baseline KOOS value	Decrease KOOS pain (KOOS symptom and quality of life show similar regression coefficients)	β 0.49 (0.43–0.59)	+
	Pisters [27] 2012	Per cm increase VAS^a^	Increase in self-reported limitations (WOMAC-PF)	β 5.99 (2.90–12.4)	+
	Sharma [34] 2003	Per 20 mm VAS increase	Progressing/remaining in poor WOMAC-PF quintiles	OR 1.48 (1.12–1.95)	+
	Riddle [30] 2012	NRS of past 30 days^a^	Knee joint surgery	RR 1.12 (1.02–1.22)	+
Painful knee flexion	Riddle [30] 2012	Yes vs no	Knee joint surgery	RR 1.58 (1.04–2.39)	+
(N = 4,670)					
Joint line tenderness	Holla [21] 2014	Yes vs no	Poor vs good outcome WOMAC-PF trajectory	OR 2.63 (1.38–5.02)	+
(N = 5,367)	Riddle [30] 2012	Yes vs no	Knee joint surgery	RR 0.71 (0.43–1.18)	o
Flexion contracture	Riddle [30] 2012	Knee flexion contracture (°)	Knee joint surgery	RR 1.06 (1.02–1.11)	+
(N = 4,670)					
Knee ROM	Holla [21] 2014	Active ROM in degrees	Poos vs good outcome WOMAC-PF trajectory	OR 0.96 (0.93–1.00)	–
(N = 913)	Pisters [27] 2012	Mean extension	Increase in performance-based limitations (TWT)	β 0.92 (0.86–0.98)	–
Hand grip strength (muscle strength)	Muraki [25] 2012	Per 1 kg strength increase	Incident knee pain at follow-up (baseline K/L ≥2)	OR 1.00 (0.98–1.02)	o
(N = 1,313)					
Quadriceps strength	Amin [10] 2009	Low vs middle	Increase in knee specific VAS pain score	Not provided	o
(N = 5,151)		vs high strength	Increase in self-reported limitations (WOMAC-PF)	Not provided	o
	Pisters [27] 2012	Continuous in Newton/kg	Increase in self-reported limitations (WOMAC-PF)	β 0.11 (0.01–1.36)	o
			Increase in performance-based limitations (TWT)	β 0.60 (0.37–1.03)	o
	Riddle [30] 2012	Normalized to bodyweight	Knee joint surgery	RR 0.79 (0.65–0.96)	–
Bone marrow lesions/edema (BMLs/BME)	Roemer [32] 2014	≥2 subregions vs 0–1	Knee joint surgery	OR 4.00 (1.75–9.16)	+
		Grade ≥1 vs grade 0		OR 4.00 (0.85–18.84)	o
	Scher [33] 2008	Global BME vs none	Knee joint surgery	OR 15.2 (2.38–97.1)	+
(N = 580)	Tanamas [35] 2010	BMLs, present vs absent	Knee joint surgery	OR 1.57 (1.04–2.35)	+
		Medial BMLs vs absent	Knee joint surgery	OR 1.78 (1.16–2.74)	+
		Lateral BMLs vs absent	Knee joint surgery	OR 0.82 (0.43–1.54)	o
Subchondral bone cysts (MRI)	Tanamas [36] 2010	Medial, per grade of severity	Knee joint surgery	OR 1.99 (1.01–3.90)	+
		Lateral, per grade of severity	Knee joint surgery	OR 0.96 (0.48–1.94)	o
(N = 109)					
Cartilage loss (MRI)	Cicuttini [14] 2004	Rate 3–8 % per annum	Knee joint surgery	OR 2.3 (0.4–12.2)	o
(N = 681)		Rate >8 % per annum		OR 7.1 (1.4–36.5)	+
	Eckstein [17] 2013	Change in cMFTC.ThC	Knee joint surgery	Not provided	+
		Change in MFTC.ThC		Not provided	+
		Change in LFTC.ThC		Not provided	o
	Roemer [32] 2014^b^	Grade 3 vs <3 (whole knee)		OR 4.00 (2.23–7.18)	+
		Grade 3 vs 0 (MFTC)	Knee joint surgery	OR 3.01 (1.52–5.95)	+
		Grade 3 vs 0 (LFTC)		OR 1.69 (0.94–3.02)	o
	Scher [33] 2008	≥50 % vs <50 % loss	Knee joint surgery	OR 2.06 (0.74–5.70)	o
Meniscal extrusion (MRI)	Roemer [32] 2014	≥5 mm vs <5 mm (medial)	Knee joint surgery	OR 1.00 (0.60–1.67)	o
		≥5 mm vs <5 mm (lateral)		OR 1.42 (0.54–3.75)	o
(N = 398)					
Meniscal damage (MRI)	Roemer [32] 2014	Grade 6–8 vs 0–1 (medial)	Knee joint surgery	OR 1.84 (1.13–2.99)	o
(N = 398)		Grade 6–8 vs 0–1 (lateral)		OR 1.10 (0.68–1.77)	o
Anterior cruciate ligament tear	Amin [11] 2008	Complete tear on MRI	Increase in knee-specific VAS pain score	Not provided	o
		Complete tear on MRI	Increase in self-reported limitations (WOMAC-PF)	Not provided	o
(N = 265)					
Synovitis	Roemer [32] 2014	Infrapatellar fat pad on MRI	Knee joint surgery	OR 2.17 (1.33–3.56)	+
(N = 764)	Hill [19] 2007	Infrapatellar fat pad on MRI	Increase in knee-specific VAS pain score	β 4.89 (0.42–9.36)	+
		Intercondylar on MRI		β 5.74 (0.34–11.14)	+
		Suprapatellar on MRI		β 3.35 (–0.34 to 7.05)	o
	Conaghan [16] 2010	Present on US	Knee joint surgery	HR 1.54 (0.95–2.50)	o
Joint effusion	Conaghan [16] 2010	Present on US	Knee joint surgery	HR 3.06 (2.00–4.69)	+
(N = 5,979)	Riddle [29] 2009	Positive bulge sign	Knee joint surgery	OR 2.53 (1.13–5.56)	+
	Riddle [30] 2012	Positive bulge sign	Knee joint surgery	RR 1.58 (1.04–2.40)	+
	Roemer [32] 2014	Present on MRI (grade 1–3)	Knee joint surgery	OR 4.75 (2.55–8.85)	+
Trabecular bone texture	Podsiadlo [28] 2014	FD_mean_ on FSA, medial	Knee joint surgery	OR 0.23 (0.06–0.82)	+
(N = 114)		FD_mean_ on FSA, lateral		OR 0.33 (0.09–1.22)	o
C2C (serum)	Berry [12] 2009	High level vs low	Knee joint surgery	OR 1.01 (0.94–1.08)	o
(N = 117)					
COMP (serum)	Berry [12] 2010	High level vs low	Knee joint surgery	OR 0.77 (0.15–3.81)	o
(N = 117)					
PIIANP (serum)	Berry [12] 2010	Natural log baseline levels	Knee joint surgery	OR 0.28 (0.10–0.93)	–
(N = 117)					
ARGS (synovial)	Larsson [23] 2012	Baseline level ARGS > follow-up level ARGS	≥10 units progression KOOS Pain	OR 3.66 (1.01–13.2)	+
(N = 74)			≥10 units progression KOOS Function of daily living	OR 1.11 (0.26–4.80)	o

*Statistically significant association of the determinant with OA progression: + positive association, – negative association, o no association (adjusted for age and sex if applicable). ^a^Assessed at baseline. ^b^Similar results found when measuring compartment cartilage thickness. *ARGS* aggrecan neoepitope amino acid sequence, *β* regression coefficient, *CI* confidence interval, *(c)MFTC.ThC* (central) medial femorotibial compartment cartilage thickness (in mm), *FD*_*mean*_ mean fractal dimension, *FSA* fractal signal analysis, *C2C* collagen type-II cleavage, *COMP* cartilage oligomeric matrix protein, *HR* hazard ratio, *JSN* joint space narrowing, *JSW* joint space width, *K/L* Kellgren and Lawrence score, *KOOS* Knee Injury and Osteoarthritis Outcome Score, *LFTC* lateral FTC, *N* combined sample size, *MRI* magnetic resonance imaging, *NRS* Numeric Rating Scale, *OA* osteoarthritis, *OR* odds ratio, *PIIANP* N-propeptide of type IIA collagen, *ROM* range of motion, *RR* relative risk, *TWT* Timed Walking Test, *US* ultrasonography, *VAS* Visual Analogue Scale, *WOMAC-PF* physical function scale of the Western Ontario and McMaster osteoarthritis index

#### Patient characteristics

Patient characteristics are show in Table 3. Two studies found significant positive associations between age and cOA progression [29, 30]. One study [25] found no association and three studies [15, 20, 21], two of which are from the same cohort, found a slight negative association.

Muraki et al. found no association between gender and cOA progression [25]. Collins et al. found significant associations for low moderate, high moderate and severe pain trajectories compared to no pain trajectory (not all data in Table 3) [15].

Holla et al. determined a significantly increased risk for symptom progression in non-western participants compared to western participants [20]. Collins et al. found similar results comparing whites with non-whites [15]. They also found increased risks for cOA progression for a lower education level, as did Riddle et al. [29].

Six authors performed analyses determining the association between body mass index (BMI) and cOA progression [15, 20, 21, 25, 29, 34]. Five out of six analyses found statistically significant positive associations [15, 20, 21, 25, 29]; Sharma et al. found no association [34].

Riddle and Stratford investigated the influence of body weight change (either a reduction or gain) and cOA progression [31]. They found that only at least 10 % change in bodyweight significantly influences the risk of cOA progression. Henriksen et al. found no association for change in peak knee joint compressive forces and cOA progression [18]. A decrease in peak knee force (or unloader) was defined as decrease in body mass, unchanged walking speed, and a decreased knee extensor moment.

Five authors studied co-morbidity as a determinant for cOA progression [15, 20, 21, 27, 37]. Holla et al. found no association for co-morbidity count in one study [20], but found an association in another study within the same cohort [21]. Collins et al. [15], Pisters et al. [27] and van Dijk et al. [37] found that an increase in co-morbidity count led to a significant increase in cOA progression.

Collins et al. found that depression (Center for Epidemiologic Studies Depression Scale ≥16) increased the risk for a unfavorable pain trajectory [15]. Sharma et al. studied the association between a mental health survey score and the progression of limitations in physical functioning [34]. A higher mental health score (i.e., better mental health) was associated with a decreased risk for a poor outcome on the Physical function scale of the Western Ontario and McMaster osteoarthritis index (WOMAC-PF) scale. Riddle et al. found a reversed association per unit of the Short form survey instrument for the mental component summary (SF-12 MCS) score and knee joint surgery [30].

van Dijk et al. [37] and Holla et al. [21] reported a favorable effect of high vitality on cOA progression. Alschuler et al. found associations for the coping strategy catastrophizing and praying or hoping (not all data presented in Table 3) [9]. Holla et al. found no association for frequent use of the pain coping strategy distraction, but found a significant association for infrequent use of the pain coping strategy worrying [20]. Pisters et al. [27] and Holla et al. [21] found significant associations for cOA progression when applying the pain coping strategy resting (i.e., avoidance of activity).

Holla et al. found an association between morning stiffness of the knee joint (<30 minutes) and a poor outcome on the WOMAC-PF scale [20]. Muraki et al. found a significant association between previous knee injury and incident knee pain at follow-up in patients with K/L ≥2 at baseline [25].

Riddle et al. determined a significant association for participants with a history of knee surgery, but no associations for history of hip replacement surgery [30]. Riddle et al. [30] and Lapane et al. [22] found no associations for frequent medication use. Laslett et al. found an association between bisphosphonate use and decrease in the numeric rating scale (NRS) after 3 and 4 years, but not after 5 years; however, medication compliance did drop remarkably in this study by the fifth year [24]. They found no association for WOMAC scores. Yang et al. found no clinically significant differences between users and non-users of glucosamine and/or chondroitin in WOMAC pain, stiffness or function [38].

#### Disease characteristics

Disease characteristics are show in Table 4. Multiple studies were performed determining the associations for baseline radiographic or clinical severity of OA [9, 13, 15, 21, 26, 27, 30, 34]. Bruyere et al. found an increased risk for knee joint surgery in patients with an increased rate of joint space narrowing per 3 years [13]. Collins et al. [15], Riddle et al. [30] and Oak et al. [26] found significant associations for both baseline radiographic severity and baseline pain. Alschuler et al. found associations for baseline pain and function scores [9]. Pisters et al. found a significant association between baseline pain intensity and self-reported limitations on the WOMAC-PF scale [27]. Sharma et al. determined a significantly positive association for baseline VAS pain score [34]. Holla et al. found significant associations for baseline osteophytosis and NRS for pain [21].

Riddle et al. found that a painful knee flexion and a flexion contracture were significantly associated with future knee joint surgery, but knee joint line tenderness was not associated [30]. Holla et al. did find an association for bony tenderness [21]. Pisters et al. [27] and Holla et al. [21] reported that a larger baseline knee range of motion (ROM) was significantly associated with less knee cOA progression. Muraki et al. studied hand grip strength in participants with knee cOA progression, as an indication of general muscle strength, and found no significant associations [25].

Three authors studied the association between quadriceps strength and cOA progression [10, 27, 30]. Only one study found an association, describing significantly lower mean baseline quadriceps strength in patients with cOA progression [30].

Scher et al. found a significant association for MRI-detected global bone marrow edema [33]. Roemer et al. found an association in knees with more than two subregions with bone marrow lesions (BMLs), but no association when scoring BMLs [32]. Tanamas et al. investigated the association for BMLs in the tibiofemoral joint [35]. They found significant associations for the total presence of BMLs and for medial BMLs. The association for lateral BMLs was not statistically significant. The authors also found an association for MRI-detected subchondral bone cysts in the medial tibiofemoral compartment, but not for the lateral compartment [36].

MRI-detected cartilage loss and the risk of cOA progression was studied by four authors [14, 17, 32, 33]. Cicuttini et al. reported a significant association between cartilage loss rate >8 % per annum and knee joint surgery [14]. Eckstein et al. found significant positive associations for increased cartilage thickness loss in the medial tibiofemoral compartment [17]. They found no significant association in the lateral compartment. Similar significant associations were found in their analyses when calculating the percentage denuded area of subchondral bone in the medial compartment (data not presented in this review). Roemer et al. found elevated risks in knees that exhibited ≥2 compartments with severe cartilage loss on MRI [32]. Scher et al. found no significant associations [33].

Roemer et al. found an association with knee joint surgery in knees with MRI-detected medial meniscus maceration, but not for lateral maceration or meniscal extrusion [32]. Amin et al. found no significant association for MRI-detected anterior cruciate ligament tear [11].

Hill et al. found significant correlations for the presence of MRI-detected infrapatellar and intercondylar synovitis at baseline [19]. The correlation for suprapatellar synovitis was non-significant. Conaghan et al. found no association for synovitis detected by ultrasonography (US) [16]. They did report a significant association for US-detected joint effusion [16]. Riddle et al. also reported significant associations for clinically detected joint effusion (positive bulge sign) [29, 30]. Roemer et al. found associations for both MRI-detected effusion and synovitis [32].

Podsiadlo et al. found that an increase in overall roughness of medial tibial trabecular bone texture, or fractal dimension, detected on fractal signal analysis led to a risk reduction for knee joint surgery [28]. All other fractal dimension regions of interest showed non-significant associations (data not presented in Table 4).

Berry et al. studied the associations between three serum markers and cOA progression [12]. They found no association for serum collagen type-II cleavage (C2C) or for serum levels of cartilage oligometric matrix protein (COMP). They did find that serum N-propeptide of type II collagen was associated with a significantly reduced risk for knee joint replacement.

Larsson et al. found an association between synovial fluid aggrecan neoepitope amino acid sequence (ARGS) levels and pain progression, but not between ARGS levels and function of daily living [23].

### Best evidence synthesis

Pooling was not possible due to heterogeneity, hence we were forced to apply a best evidence synthesis (Table 5), which demonstrated strong evidence that age, ethnicity, BMI, co-morbidity count, MRI-detected infrapatellar synovitis, joint effusion and baseline OA severity (both radiographic and clinical) are associated with the progression of clinical knee OA. There was moderate evidence showing that education level, vitality, pain-coping subscale resting, MRI-detected medial femorotibial cartilage loss and general BMLs are associated with knee cOA progression.

**Table 5:** Results from the best evidence synthesis: associations with clinical knee OA progression

Determinants	Level of evidence
Age, ethnicity, BMI, co morbidity count, MRI-detected infrapatellar synovitis, joint effusion and baseline OA severity (radiographic and clinical)	Strong evidence for association
Education level, vitality, pain coping subscale resting, MRI-detected medial femorotibial cartilage loss and general BMLs	Moderate evidence for association
Pain coping subscales worrying, hoping and catastrophizing, knee injury, knee surgery, bisphosphonate usage, painful knee flexion, flexion contracture, knee ROM, medial BMLs, medial subchondral bone cysts and medial trabecular bone texture	Limited evidence for association
Knee compression force, pain coping subscale distraction, morning stiffness, pain medication usage, glucosamine or chondroitin usage, hip replacement surgery, joint line tenderness, muscle strength, lateral BMLs, lateral subchondral bone cysts, lateral femorotibial cartilage loss, meniscal extrusion or damage, anterior cruciate ligament tear, intercondylar or suprapatellar synovitis on MRI, synovitis on US, lateral trabecular bone texture serum markers C2C and COMP	Limited evidence for no association
Gender, mental health, bisphosphonate usage, joint line tenderness, quadriceps strength, MRI-detected whole knee cartilage loss and synovial marker ARGS	Conflicting evidence
Bodyweight change	Inconclusive evidence
*ARGS* aggrecan neoepitope amino acid sequence, *BMI* body mass index, *BML*, bone marrow lesion, *C2C* collagen type-II cleavage, *COMP* cartilage oligomeric matrix protein, *MRI* magnetic resonance imaging, *OA* osteoarthritis, *ROM* range of motion, *US* ultrasonography	

There is limited evidence that pain coping subscales worrying, hoping and catastrophizing, knee injury, knee surgery, bisphosphonate usage, painful knee flexion, flexion contracture, knee ROM, medial BMLs, medial subchondral bone cysts and medial trabecular bone texture are associated with the cOA progression. There is also limited evidence that there is no association between clinical knee OA progression and knee compression force, pain coping subscale distraction, morning stiffness, pain medication usage, glucosamine or chondroitin usage, hip replacement surgery, joint line tenderness, muscle strength, lateral BMLs, lateral subchondral bone cysts, lateral femorotibial cartilage loss, meniscal extrusion or damage, anterior cruciate ligament tear, synovitis other than infrapatellar, lateral trabecular bone texture, and serum markers C2C and COMP.

Conflicting evidence was found for the associations between clinical knee OA progression and gender, mental health, bisphosphonate usage, joint line tenderness, quadriceps strength, MRI-detected whole knee cartilage loss and synovial marker ARGS. There was inconclusive evidence for the associations found between cOA progression and bodyweight change.

### Sensitivity analysis

No conclusions were influenced or altered by differences in sample size or follow-up duration. When analyzing the definitions for cOA progression, we found irregularity in the strong evidence found for age as a risk factor. Five out of six studies found significant associations with clinical knee OA progression. Three of these five associations were negative associations (i.e., lower baseline age resulted in higher risk for progression); the remaining two associations were positive associations. However, these two positive associations defined cOA progression as knee joint surgery, where the other three negative associations defined cOA progression by pain or function scores. By splitting these definitions of cOA progression, the evidence for age would remain strong, but lower age would be labeled as a risk factor for more severe symptom progression and higher age would be labeled as a risk factor for knee joint surgery due to OA.

## Discussion

There is strong evidence that age, ethnicity, BMI, co-morbidity count, MRI-detected infrapatellar synovitis, joint effusion and both radiographic and clinical baseline OA severity are predictive for clinical knee OA progression. However, for the majority of studied determinants in our review the evidence is limited, conflicting or inconclusive.

More precise estimates of associations could have been given if pooling was possible, but this was not feasible due to large variation in criteria for defining disease (progression). Six different criteria were used for inclusion of OA (see Table 2) and nine definitions were applied for cOA progression (see Tables 3 and 4). Furthermore, variables under study were measured differently (continuous, dichotomous, or categorical with varying cut-off points).

Age has previously been recognized as a risk factor for progression on symptomatic knee OA by van Dijk et al. [39]. In this 2006 review determining prognostic factors for functional status in knee OA, the authors presented similar evidence on age as a risk factor. Oddly enough, as presented in our sensitivity analysis, a lower baseline age is associated with an increased risk of symptom progression, whereas a higher baseline age results in an increased risk for undergoing knee joint surgery due to knee OA. This inverse association is not properly understood yet and should be explored in future studies.

Overweight has previously been recognized as a risk factor for incident knee OA [40, 41]. The evidence for an association between overweight and progression of radiographic knee OA remains conflicting [2, 42], but this review shows strong evidence for the association between BMI and symptom progression which is in line with earlier findings by van Dijk et al. [39].

An association between knee pain and joint effusion has been found before in cross-sectional analysis, but the exact pathophysiology needs to be better understood [43]. Previous reviewers found similar results for MRI-detected effusion or synovitis, but these results are based on cross-sectional studies or on the same longitudinal studies included in this review [44]. Our results show that joint effusion, which is relatively easy and cheap to ascertain in primary care by physical examination or US, seems to be a strong predictor of symptom progression and it underlines the importance of proper physical examination.

High baseline OA severity scores were associated with clinical knee OA progression. It seems logical that subjects with initial severe symptoms are prone to symptom progression, but there is a discrepancy in the evidence for radiographic OA severity and symptom severity [45]. In this 2009 review of the (mainly cross-sectional) literature the authors however state that many studies have not used X-ray views of all three compartments of the knee, which could have contributed to an underestimation of the association between radiographic knee OA and clinical symptoms [45].

We found notable overlap with the evidence for clinical hip OA progression in two large reviews, defining clinical hip OA progression as total hip arthroplasty (THA) [5, 46]. The authors presented conflicting evidence regarding age and gender, but consistent evidence for associations between both radiographic and clinical baseline severity with THA. Moreover, there was limited evidence for an association between BMI and no association between serum COMP with THA.

A point of discussion could be our choice of outcome measure inclusion, i.e., including and comparing pain progression, physical function decline and knee joint surgery. Although these measures are not the same, there exists a strong correlation between these outcomes. Moreover, presenting these results together provides a clear overview of all existing evidence regarding symptomatic knee OA progression. One observation that strongly becomes apparent is the lack of studies investigating risk factors for pain progression in knee OA, when pain has shown to be the number one complaint in patients with (knee) OA [1]. On the other hand, pain is an important indication for undergoing knee joint surgery, which will be further addressed below.

Our study may have limitations. Firstly, limitations to reviewing observational studies on disease progression have been addressed, stating that, unlike randomized trials, observational studies of pre-existing disease are subject to various biases that may account for discrepancies found between risk factors for incidence and progression [47]. The hypothesis is that risk factors may exist for progressive knee OA, but that flaws in study design and the measure of disease progression may prevent true detection of risk factors [47]. Secondly, some outcome measures were only assessed once at follow-up, which consequently could have led to an incorrect assessment of true clinical OA progression. Pain and physical limitations due to OA fluctuate over time, hence multiple outcome measure assessments during follow-up would give a better depiction of disease progression [48]. Finally, using knee joint surgery as an outcome measure for clinical knee OA progression might lead to discussion, considering orthopedic surgeons would generally not operate on a knee that shows no sign of (progressed) radiographic OA. However, studies have shown that a key indicator for undergoing knee joint surgery in patients with knee OA is pain or disability [30, 49].

When comparing our results to the results found in the review by Belo et al. [2], substantial differences in prognostic factors for cOA progression can be detected compared to risk factors for radiographic progression of knee OA. Belo et al., for instance, found strong evidence for no association for gender and quadriceps strength, when we found conflicting evidence for both determinants. Moreover, there are differences in the number of investigated possible risk factors. For example, Belo et al. found strong evidence for the association of serum levels of hyaluronic acid with radiographic knee OA progression, when no articles investigating hyaluronic acid were included in this current review. The abovementioned underlines the importance of distinguishing (possible) risk factors for clinical knee OA progression from (possible) risk factors for radiographic knee OA progression.

More research is needed on the true relationship between prognostic factors for symptomatic knee OA progression, especially regarding factors where conflicting, limited or inconclusive evidence was presented. It would be very convenient if a physician was enabled to closely monitor patients with symptomatic knee OA who are at high risk for rapid or severe symptom progression. Moreover, potential risk factors which can be modified at an early stage of the disease, i.e., pain coping strategies or quadriceps strength, could prove to have substantial benefit in the treatment of patients with knee OA. In addition, the etiology and pathophysiology of radiographic OA features, joint effusion, BMLs and subchondral cysts in knee OA and their relation with clinical symptoms longitudinally should be further explored.

## Conclusions

In conclusion, we have summarized the available evidence of prognostic factors for clinical knee OA progression. A large variety in definitions of clinical knee OA (progression) remains, which unfortunately makes it impossible to properly summarize the evidence through meta-analyses. More research on prognostic factors for knee OA is needed using symptom progression as an outcome measure. There are remarkably few studies that study pain progression in patients with knee OA.

## Abbreviations

ACR: American College of Rheumatology
ARGS: Aggrecan neoepitope amino acid sequence
BMI: Body mass index
BML: Bone marrow lesion
C2C: Collagen type-II cleavage
CI: Confidence intervals
cOA: Clinical osteoarthritis
COMP: Cartilage oligomeric matrix protein
HR: Hazard ratios
K/L: Kellgren and Lawrence score
MRI: Magnetic resonance imaging
NRS: Numeric rating scale
OA: Osteoarthritis
OARSI: Osteoarthritis Research Society International Atlas
OR: Odds ratios
ROM: Range of motion
RR: Relative risks
SF-12 MCS: Short form survey instrument for the mental component summary
THA: Total hip arthroplasty
US: Ultrasonography
VAS: Visual analogue scale
WOMAC: Western Ontario and McMasters osteoarthritis index
WOMAC-PF: Physical function scale of the Western Ontario and McMaster osteoarthritis index
